# Supplementary material for: Brigatinib for Pretreated, ALK-Positive, Advanced Non-Small-Cell Lung Cancers: Long-Term Follow-Up and Focus on Post-Brigatinib Lorlatinib Efficacy in the Multicenter, Real-World BrigALK2 Study
Source: Cancers (Basel). 2022 Mar 30;14(7):1751. doi: 10.3390/cancers14071751 (PMC8997056; doi:10.3390/cancers14071751)
Supplement: Supplementary file 1 [file cancers-14-01751-s001.zip › cancers-1604794-supplementary.pdf]

cancers

MDPI

Supplementary Materials: Brigatinib for Pretreated, ALK-Positive, Advanced Non-Small-Cell Lung Cancers: Long-Term Follow-Up and Focus on Post-Brigatinib Lorlatinib Efficacy in the Multicenter, Real-World BrigALK2 Study

Renaud Descourt, Maurice Pérol, Gaëlle Rousseau-Bussac, David Planchard, Bertrand Mennecier, Marie Wislez, Jacques Cadranel, Alexis Benjamin Cortot, Florian Guisier, Loïck Galland, Pascal Do, Roland Schott, Éric Dansin, Jennifer Arrondeau, Jean-Bernard Auliac, Margaux Geier and Christos Chouaïd

Table S1. Treatment lines and agent sequences administered before brigatinib.

**Treatment lines before brigatinib**	***n* = 174***
Sequence of agent(s) given	
**One line**	**15**
Crizotinib	13
Ceritinib	2
**Two lines**	**44**
Crizotinib then ceritinib	36
Crizotinib then chemotherapy	1
Chemotherapy then ceritinib	2
Crizotinib then alectinib	1
Chemotherapy then crizotinib	4
**Third lines**	**74**
Chemotherapy, crizotinib then ceritinib	49
Chemotherapy, crizotinib then alectinib	5
Chemotherapy, chemotherapy then crizotinib	2
Crizotinib, chemotherapy then ceritinib	4
Chemotherapy, chemotherapy then ceritinib	1
Crizotinib, ceritinib then chemotherapy	8
Alectinib, crizotinib then ceritinib	1
Chemotherapy, ceritinib then crizotinib	2
Chemotherapy, ceritinib then lorlatinib	1
Crizotinib, alectinib then ceritinib	1
**Four lines**	**31**
Chemotherapy, chemotherapy, chemotherapy then crizotinib	1
Chemotherapy, chemotherapy, crizotinib then chemotherapy	5
Chemotherapy, chemotherapy, crizotinib then ceritinib	1
Chemotherapy, crizotinib, chemotherapy then crizotinib	3
Chemotherapy, crizotinib, chemotherapy then ceritinib	3
Chemotherapy, crizotinib, ceritinib then chemotherapy	3
Chemotherapy, crizotinib, ceritinib then alectinib	3
Chemotherapy, crizotinib, ceritinib then lorlatinib	1
Chemotherapy, crizotinib, ceritinib then another	1
Crizotinib, chemotherapy, crizotinib then ceritinib	1
Crizotinib, chemotherapy, ceritinib then chemotherapy	4
Crizotinib, chemotherapy, ceritinib then chemotherapy	1

Crizotinib, ceritinib, chemotherapy then chemotherapy	1
Crizotinib, ceritinib, chemotherapy then crizotinib	1
Crizotinib, ceritinib, lorlatinib then chemotherapy	1
Ceritinib, chemotherapy, crizotinib then ceritinib	1
**Five lines**	**10**
Chemotherapy, chemotherapy, crizotinib, ceritinib then chemotherapy	1
Chemotherapy, crizotinib, ceritinib, ceritinib then chemotherapy	1
Chemotherapy, crizotinib, ceritinib, immunotherapy then ceritinib	2
Chemotherapy, crizotinib, ceritinib, chemotherapy then crizotinib	1
Chemotherapy, crizotinib, ceritinib, chemotherapy then lorlatinib	1
Chemotherapy, crizotinib, ceritinib, immunotherapy then chemotherapy	1
Crizotinib, chemotherapy, chemotherapy, ceritinib then chemotherapy	1
Crizotinib, chemotherapy, alectinib, ceritinib then chemotherapy	1
Crizotinib, chemotherapy, alectinib, chemotherapy then chemotherapy	1

* 9 patients had more than 5 lines of treatments and were not described here.
